# LETTER TO THE EDITOR S-Plasty Method for Secondary Scar Revision After Flap Surgery

**Published:** 2011-06-27

**Authors:** Shimpei Ono, Sandeep J. Sebastin, Rei Ogawa, Hiko Hyakusoku

**Affiliations:** ^a^Department of Plastic, Reconstructive and Aesthetic Surgery, Nippon Medical School, Tokyo, Japan; ^b^Department of Hand and Reconstructive Microsurgery, National University Health System, Singapore

Dear Sir,

We would like to present the use of the S-plasty method for revising the inset of a flap. Occasionally, the inset of a flap results in a depressed scar at the junction between the flap and the normal skin. This scar is further accentuated in a bulky flap. An S-plasty was designed over the depressed portion of the scar (Fig. 1). The S-plasty involves making a series of small-wave like incisions that are approximately 0.5 to 1.0 cm in length. The scar measured 6.5 cm preoperatively and the immediate postoperative length was 8 cm. Figure 2 shows the late postoperative appearance.

The lengthening in an S-plasty is based on the so-called “accordion effect” whereby a curvilinear “S” is stretched out linearly. The degree of lengthening that occurs in an S-plasty can be calculated by a mathematical formula (Fig. 3). If the length of the scar is “L,” the length of the revised scar “L1” will be approximately 1.2 × L.

The W-plasty is also based on the “accordion effect,” whereby the limbs of the W spread apart to increase length of the scar thus relieving tension. However, this method leaves noticeable zig-zag scars and requires the resection of normal skin (Fig. 4). To overcome these problems, Hyakusoku et al^1^ introduced the “S-plasty” method in 2004. This method maintains the lengthening of the scar by the accordion effect, avoids the zig-zag scars of the W-plasty, and requires minimal resection of normal skin. We have previously used this technique to treat long linear hypertrophic scars of the suprapubic lesion with excellent results.

## Figures and Tables

**Figure 1:**
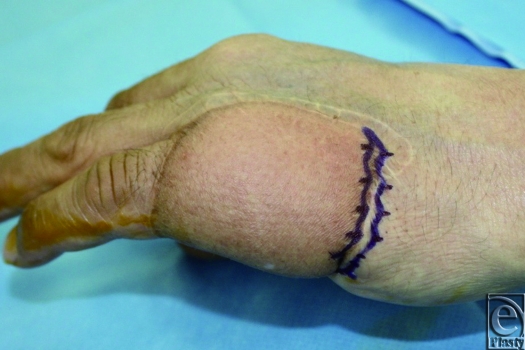
Design of S-plasty over the depressed scar at the junction of abdominal flap and normal skin.

**Figure 2:**
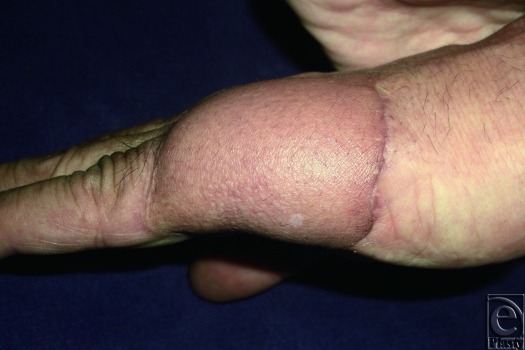
Postoperative appearance at 6 months.

**Figure 3:**
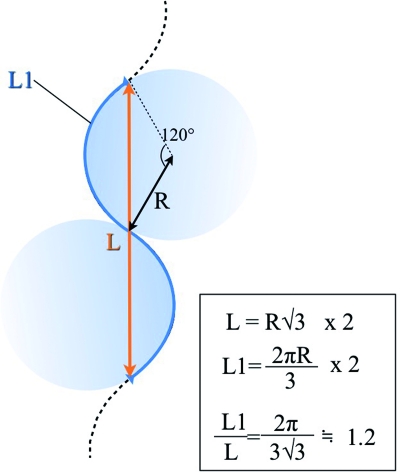
Maximal lengthening of scar obtained in a S-plasty.

**Figure 4:**
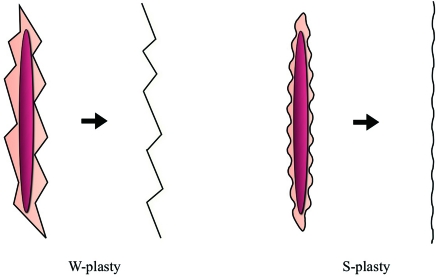
Comparison of W-plasty and S-plasty techniques.
